# Effectiveness of COVID-19 XBB.1.5 monovalent mRNA vaccine in Korea: interim analysis

**DOI:** 10.3389/fimmu.2024.1382944

**Published:** 2024-05-13

**Authors:** Eliel Nham, Jang Wook Sohn, Won Suk Choi, Seong-Heon Wie, Jacob Lee, Jin-Soo Lee, Hye Won Jeong, Joong Sik Eom, Yu Jung Choi, Hye Seong, Jin Gu Yoon, Ji Yun Noh, Joon Young Song, Hee Jin Cheong, Woo Joo Kim

**Affiliations:** ^1^ Division of Infectious Diseases, Department of Internal Medicine, Korea University College of Medicine, Seoul, Republic of Korea; ^2^ Vaccine Innovation Center-Korea University (KU) Medicine (VIC-K), Seoul, Republic of Korea; ^3^ Division of Infectious Diseases, Department of Internal Medicine, St. Vincent’s Hospital, College of Medicine, The Catholic University of Korea, Suwon, Republic of Korea; ^4^ Division of Infectious Diseases, Department of Internal Medicine, Kangnam Sacred Heart Hospital, Hallym University College of Medicine, Seoul, Republic of Korea; ^5^ Division of Infectious Diseases, Department of Internal Medicine, Inha University School of Medicine, Incheon, Republic of Korea; ^6^ Department of Internal Medicine, Chungbuk National University College of Medicine, Cheongju, Republic of Korea; ^7^ Division of Infectious Diseases, Department of Internal Medicine, Gil Medical Center, Gachon University College of Medicine, Incheon, Republic of Korea

**Keywords:** COVID-19, SARS-CoV-2, XBB.1.5, Omicron, vaccine, effectiveness

## Abstract

As coronavirus disease-2019 (COVID-19) becomes an endemic disease, the virus continues to evolve and become immunologically distinct from previous strains. Immune imprinting has raised concerns about bivalent mRNA vaccines containing both ancestral virus and Omicron variant. To increase efficacy against the predominant strains as of the second half of 2023, the updated vaccine formulation contained only the mRNA of XBB.1.5 sublineage. We conducted a multicenter, test-negative, case-control study to estimate XBB.1.5 monovalent vaccine effectiveness (VE) and present the results of an interim analysis with data collected in November 2023. Patients who underwent COVID-19 testing at eight university hospitals were included and matched based on age (19-49, 50-64, and ≥65 years) and sex in a 1:1 ratio. VE was calculated using the adjusted odds ratio derived from multivariable logistic regression. Of the 992 patients included, 49 (5.3%) received the XBB.1.5 monovalent vaccine at least 7 days before COVID-19 testing. Patients with COVID-19 (cases) were less likely to have received the XBB.1.5 monovalent vaccine (case 3.5% vs. control 7.2%, p=0.019) and to have a history of COVID-19 within 6 months (2.2% vs. 4.6%, p=0.068). In contrast, patients with COVID-19 were more likely to be healthcare workers (8.2% vs. 3.0%, p=0.001) and to have chronic neurological diseases (16.7% vs. 11.9%, p=0.048). The adjusted VE of the XBB.1.5 monovalent mRNA vaccine was 56.8% (95% confidence interval: 18.7-77.9%). XBB.1.5 monovalent mRNA vaccine provided significant protection against COVID-19 in the first one to two months after vaccination.

## 1 Introduction

Since its first emergence in late 2019, the ever-changing nature of the severe acute respiratory syndrome coronavirus 2 (SARS-CoV-2) has made previous immunity acquired through infection or vaccines based on ancestral SARS-CoV-2 less effective. This immune evasion became more apparent with the emergence of the Omicron variant and its sublineages since late 2021 (1–3). To combat this, bivalent vaccines containing the mRNA of both ancestral SARS-CoV-2 and Omicron variant were administered as a booster shot in 2022. However, near-total replacement of the ancestral SARS-CoV-2 by the Omicron variant and the phenomenon of immune imprinting have raised questions about the utility of including the mRNA of the ancestral SARS-CoV-2 in future coronavirus disease 2019 (COVID-19) vaccines (4–8).

In mid-2023, the World Health Organization and the United States (US) Food and Drug Administration advised pharmaceutical companies to only include component of the XBB lineage (“monovalent”) in the updated COVID-19 vaccines for the 2023/2024 season (9, 10). XBB-monovalent vaccines were also recommended by the Advisory Committee on Immunization Practices for the corresponding season, serving as both a booster dose and primary series, for all persons aged six months or older (11).

Similar to influenza vaccines, vaccine efficacy reports are not required for these updated vaccines before authorization. Although it has become essential to monitor the effectiveness of COVID-19 vaccines, studies on this matter are scarce. To fill this knowledge gap, we aimed to investigated COVID-19 XBB.1.5 monovalent mRNA vaccine effectiveness in Korea. This is an interim analysis of data collected through a network of tertiary teaching hospitals created to monitor respiratory infectious diseases since 2011 (12).

## 2 Methods

This is a retrospective, multicenter, test-negative, case-control study. Patients aged 19 years or older who underwent COVID-19 testing at eight participating hospitals (mostly located in the Seoul Metropolitan Area) were included. Individuals who were tested for COVID-19 as part of pre-admission surveillance (i.e., without clinical or epidemiological risk factors for COVID-19) were excluded. Cases were defined as patients who tested positive for COVID-19 using reverse transcription polymerase chain reaction (RT-PCR) or a lateral flow assay with nasopharyngeal swab samples. Those that tested negative were classified as controls. The limit of detection for the RT-PCR test and RAT we used is 5,000 copies/mL and 2.5×10^1.8^ TCID_50_/mL, respectively. For SARS-CoV-2, 2.5×10^1.8^ TCID_50_/mL falls in the range between 5×10^5^–1×10^6^ copies/mL (13). The LOD value of RAT meets the WHO requirement of 100–1,000 TCID_50_/mL. For this interim analysis, data collected in November 2023 were included.

The case and control groups were matched based on age (19–49, 50–64, and ≥65 years) and sex in a 1:1 ratio. Information on demographics, COVID-19 vaccination history, recent history of COVID-19, comorbidities, symptoms, and clinical outcomes was collected through chart review. The vaccination status was verified using the National Immunization Registry of the Korean Disease Control and Prevention Agency. The vaccine was considered effective if administered at least seven days before the date of COVID-19 testing. In Korea, two mRNA vaccines were available (Comirnaty Omicron XBB.1.5, Spikevax XBB.1.5) since October 19, 2023. Comirnaty and Spikevax are mRNA vaccines manufactured by Pfizer-BioNTech (New York, NY, USA and Mainz, Germany) and Moderna (Cambridge, MA, USA), respectively. Each vaccine contains 30 μg/0.3mL and 50 μg/0.5mL of mRNA encoding the spike protein of SARS-CoV-2 Omicron XBB.1.5 sublineage. Both vaccines were administered intramuscularly.

Categorical variables were compared using the chi-square test or Fisher’s exact test. In addition to receipt of the XBB.1.5 monovalent vaccine, the following variables were included in the multivariable logistic regression: age, sex, comorbidities, receipt of bivalent vaccination in 2022, recent history of COVID-19, and being a healthcare worker. We then removed variables with p values greater than 0.15 from the multivariable model (backward selection). Multicollinearity between variables was defined as a variance inflation factor greater than 10. The goodness of fit of the regression model was examined using the Hosmer–Lemeshow test. Vaccine effectiveness (VE) was calculated using the adjusted odds ratio derived from the multivariable logistic regression (1-adjusted odds ratio). Statistical significance was defined as p < 0.05. Statistical analyses were performed using the R software (version 4.3.1; R Foundation, Vienna, Austria).

This study was reviewed and approved by the Institutional Review Board (IRB) of each participating hospital: Korea University Guro Hospital (approval no. 2022GR0360), Korea University Anam Hospital (2022AN0449), Korea University Ansan Hospital (2022AS0226), St. Vincent’s Hospital (VC22TIDI0150), Kangnam Sacred Heart Hospital (HKS 2022-07-016), Inha University Hospital (2022-07-036), Chungbuk National University Hospital (2022-08-022), and Gil Medical Center (GAIRB2022-306). The requirement for written informed consent was waived because of the retrospective nature of this study.

## 3 Results

A total of 992 patients were included in the study. Information on the baseline characteristics and clinical outcomes is summarized in **Tables 1** and **2**. Among them, 49 (5.3%) received the XBB.1.5 monovalent mRNA vaccine at least seven days before the date of COVID-19 testing. The median time since vaccination to COVID-19 testing was 18 days (range, 7–36 days; interquartile range, 12–27 days). Two hundred and four (22.1%) patients received BA.1 (n=89, 45.1%) or BA.4/5 (n=112, 54.9%) bivalent COVID-19 mRNA vaccine during the 2022/2023 season. Thirty-one patients (3.4%) reported a history of COVID-19 within six months. Those with at least one comorbidity accounted for 58.4% (n=538); diabetes mellitus (n=216, 23.4%), cardiovascular disease (148, 6.1%), and chronic neurologic disease (n=132, 14.3%) were the most common comorbidities. Out of 538, 211 (39%) was younger than 65 years and 327 (61%) was 65 years or older. Four hundred and forty-five (48.3%) patients were hospitalized and 20 (2.2%) died during hospitalization.

**Table 1:** Baseline characteristics of study population.

Characteristics	Total (n=922)	Case (n=461)	Control (n=461)	p-value
Age group (years)				>0.999
19-49	296 (32.1%)	148 (32.1%)	148 (32.1%)	
50-64	228 (24.7%)	114 (24.7%)	114 (24.7%)	
65+	398 (43.2%)	199 (43.2%)	199 (43.2%)	
Sex				>0.999
Male	420 (45.6%)	210 (45.6%)	210 (45.6%)	
Female	502 (54.4%)	251 (54.4%)	251 (54.4%)	
Receipt of XBB.1.5 monovalent mRNA vaccine	60 (6.5%)	20 (4.3%)	40 (8.7%)	0.011
≥7 days before COVID-19 testing	49 (5.3%)	16 (3.5%)	33 (7.2%)	0.019
Receipt of Omicron bivalent vaccine	204 (22.1%)	110 (23.9%)	94 (20.4%)	0.234
History of COVID-19 within six months	31 (3.4%)	10 (2.2%)	21 (4.6%)	0.068
Being a healthcare worker	52 (5.6%)	38 (8.2%)	14 (3.0%)	0.001
Reason for testing				<0.001
COVID-19-related symptoms	772 (83.7%)	360 (78.1%)	412 (89.4%)	
Close contact with patient(s)	10 (1.1%)	9 (2.0%)	1 (0.2%)	
Others	140 (15.2%)	92 (20.0%)	48 (10.4%)	
Comorbidities				
One or more	538 (58.4%)	277 (60.1%)	261 (56.6%)	
Diabetes mellitus	216 (23.4%)	114 (24.7%)	102 (22.1%)	0.392
Hypertension	104 (11.3%)	49 (10.6%)	55 (11.9%)	0.603
Cardiovascular disease	148 (16.1%)	80 (17.4%)	68 (14.8%)	0.324
Chronic lung disease	53 (5.7%)	14 (3.0%)	39 (8.5%)	<0.001
Chronic kidney disease	59 (6.4%)	31 (6.7%)	28 (6.1%)	0.788
Chronic liver disease	24 (2.6%)	12 (2.6%)	12 (2.6%)	>0.999
Chronic neurological disease	132 (14.3%)	77 (16.7%)	55 (11.9%)	0.048
Solid cancer	116 (12.6%)	59 (12.8%)	57 (12.4%)	0.921
Hematologic malignancy	19 (2.1%)	11 (2.4%)	8 (1.7%)	0.643
Use of immunosuppressant	38 (4.1%)	16 (3.5%)	22 (4.8%)	0.408
HIV infection	1 (0.1%)	1 (0.2%)	0 (0.0%)	>0.999
Pregnancy	14 (1.5%)	7 (1.5%)	7 (1.5%)	>0.999

COVID-19, coronavirus disease 2019; HIV, human immunodeficiency virus.

**Table 2:** Clinical outcomes of study population.

Characteristics	Total (n=922)	Case (n=461)	Control (n=461)
Symptoms			
Fever	472 (51.2%)	239 (51.8%)	233 (50.5%)
Cough	293 (31.8%)	157 (34.1%)	136 (29.5%)
Sputum	221 (24.0%)	109 (23.6%)	112 (24.3%)
Sore throat	143 (15.5%)	92 (20.0%)	51 (11.1%)
Rhinorrhea/Nasal congestion	105 (11.4%)	60 (13.0%)	45 (9.8%)
Dyspnea	193 (20.9%)	64 (13.9%)	129 (28.0%)
Chest discomfort	49 (5.3%)	9 (2.0%)	40 (8.7%)
Loss of smell	1 (0.1%)	1 (0.2%)	0 (0.0%)
Loss of taste	1 (0.1%)	1 (0.2%)	0 (0.0%)
Clinical outcomes			
Hospitalization	445 (48.3%)	213 (46.2%)	232 (50.3%)
Intensive care unit admission	94 (10.2%)	40 (8.7%)	54 (11.7%)
In-hospital death	20 (2.2%)	11 (2.4%)	9 (2.0%)
30-day mortality	18 (2.0%)	10 (2.2%)	8 (1.7%)

Compared to patients without COVID-19, lesser number of patients with COVID-19 received the XBB.1.5 monovalent vaccine at least seven days before the date of COVID-19 testing (case 3.5% vs. control 7.2%, p=0.019), have a history of COVID-19 within 6 months (2.2% vs. 4.6%, p=0.068), or have chronic pulmonary disease (3.0% vs. 8.5%, p<0.001). In contrast, case patients were more likely to be healthcare workers (8.2% vs. 3.0%, p=0.001) and have chronic neurological disease (16.7% vs. 11.9%, p=0.048). The adjusted VE of the XBB.1.5 monovalent mRNA vaccine was 56.8% (95% confidence interval: 18.7–77.9%, p = 0.011) (**Table 3**). The Hosmer-Lemeshow test showed that our model provided a good fit to the data (p = 0.988).

**Table 3:** Results of multivariable logistic regression analysis.

Variables	Odds ratio (95% confidence interval)	p-value
Receipt of XBB.1.5 monovalent mRNA vaccine*	0.432 (0.221–0.813)	0.011
History of COVID-19 within 6 months	0.538 (0.238–1.145)	0.119
Receipt of bivalent vaccine in 2022	1.340 (0.949–1.899)	0.097
Age group	1.063 (0.890–1.270)	0.499
Sex	1.040 (0.796–1.360)	0.773
Chronic pulmonary disease	0.361 (0.184–0.672)	0.002
Chronic neurological disease	1.433 (0.961–2.147)	0.078
Being a healthcare worker	2.894 (1.549–5.713)	0.001

COVID-19, coronavirus disease 2019.

*At least seven days before COVID-19 testing.

## 4 Discussion

We found that the XBB.1.5 monovalent mRNA vaccine conferred significant protection against COVID-19 in the first month after vaccination. To the best of our knowledge, this is the first study on XBB.1.5 monovalent VE against symptomatic COVID-19 outside the US as of February 5, 2024, at which this manuscript was submitted for publication. Our results are in line with the most recent US study, which reported 54% of early VE estimate in adults (14).

As it has become clear that COVID-19 is here to stay despite rapid development and distribution of effective vaccines, questions regarding future vaccination strategies have been raised. To improve immunogenicity against the immune-evasive Omicron variant and its sublineages, the first updated “bivalent” vaccine was developed in 2022. However, the bivalent vaccine did not appear to have better immunogenicity against the Omicron variant than the monovalent vaccine based on the ancestral SARS-CoV-2 (7, 15). This has led to the development of XBB-based monovalent vaccines in 2023 to increase the XBB antigen dose and avoid immune imprinting. To date, studies on this novel COVID-19 vaccine are limited. The only study that compared the immunogenicity of XBB.1.5 monovalent and bivalent (XBB.1.5 and BA.4/5) mRNA vaccines found that the monovalent vaccines were not significantly more immunogenic than the bivalent vaccine (16). Because this study was not powered to detect statistical significance, whether a monovalent vaccine would be a better choice in the future remains uncertain.

In addition to the controversy surrounding antigen doses in updated vaccines, there is an ongoing debate regarding the necessity of annual update of the COVID-19 vaccine itself. XBB.1.5 monovalent mRNA vaccines are immunogenic against emerging Omicron sublineages, including EG.5.1, HV.1, and JN.1 (16, 17). Our findings are consistent with this, considering HK.3 and EG.5 accounted for 87% of circulating sublineages during the study period (18). While studies conducted to date, including ours, suggested that XBB.1.5 monovalent vaccines were effective in preventing laboratory-confirmed COVID-19 or COVID-19-related hospitalization, cautious interpretation is needed because all of these studies only included the early post-vaccination period (14, 19, 20). More data from longer observation periods are required to establish future vaccination strategies.

There was a remarkable difference in the number of patients who experienced COVID-19-related symptoms between the case and control group. Symptoms that occurred more frequently in the control group were dyspnea (case 64 [14%] vs. control 129 [28%]) and chest discomfort (case 9 [2%] vs control 40 [9%]). This may be partly explained by the greater number of patients with chronic lung disease in the control group (case 14 [3%] vs. control 39 [9%]). Other respiratory pathogens such as influenza virus, respiratory syncytial virus, adenovirus and Mycoplasma pneumoniae that had been circulating in the community during the study period could have been the cause as well (21).

This study has several limitations. First, owing to the retrospective nature of the study, some confounders may have remained unadjusted. Second, we did not collect data on the exact number of prior COVID-19 vaccinations, which may have been a protective factor. However, this effect is likely to be small because 1) most Korean adults had completed primary series vaccination (96.6% were vaccinated twice and 75.2% three times as of October 28, 2022); 2) 78.6% of the Korean population had acquired natural immunity by contracting COVID-19 (due to any SARS-CoV-2 variant) as of August 31, 2023, which seems to be an undercount; and 3) neutralizing antibody levels after vaccination or infection wane after several months, making remote vaccination or infection less protective, especially less severe ones (22–25). Third, although the control group exclusively comprised symptomatic individuals, the case group included some asymptomatic persons (close contacts with COVID-19 cases), making the two groups less comparable in terms of healthcare-seeking behaviors. Fourth, the use of lateral flow assay, which is less sensitive than RT-PCR, may have overlooked some COVID-19 cases.

In conclusion, the XBB.1.5 monovalent mRNA vaccine provided significant protection against COVID-19 in the first month after vaccination. Full analysis results will be followed.

## Data availability statement

The datasets presented in this article are not readily available because of the institutional bans on the export of clinical data. Requests to access the datasets should be directed to WK, wjkim@korea.ac.kr.

## Ethics statement

The studies involving humans were approved by Institutional Review Board of: Korea University Guro Hospital, Korea University Anam Hospital, Korea University Ansan Hospital, St. Vincent’s Hospital, Kangnam Sacred Heart Hospital, Inha University Hospital, Chungbuk National University Hospital, and Gil Medical Center. The studies were conducted in accordance with the local legislation and institutional requirements The requirement for written informed consent was waived because of the retrospective nature of this study.

## Author contributions

EN: Data curation, Investigation, Formal analysis, Writing – original draft, Writing – review, Supervision. JWS: Data curation, Investigation, Writing – review. WSC: Data curation, Investigation, Writing – review. SHW: Data curation, Investigation, Writing – review. JL: Data curation, Investigation, Writing – review. JSL: Data curation, Investigation, Writing – review. HWJ: Data curation, Investigation, Writing – review. JSE: Data curation, Investigation, Writing – review. YJC: Investigation, Writing – review. HS: Investigation, Writing – review. JGY: Investigation, Writing – review. JYN: Investigation, Writing – review. JYS: Conceptualization, Methodology, Investigation, Writing – original draft, Writing – review, Supervision. HJC: Investigation, Writing – review. WJK: Conceptualization, Investigation, Writing – review, Supervision.
